# Evolving Epidemiology and Antibiotic Resistance in Enteric Fever: A Comprehensive Review

**DOI:** 10.7759/cureus.63070

**Published:** 2024-06-24

**Authors:** Khadija Hamdulay, Rajendra Rawekar, Ashwini Tayade, Sunil Kumar, Sourya Acharya

**Affiliations:** 1 Medicine, Jawaharlal Nehru Medical College, Datta Meghe Institute of Higher Education and Research, Wardha, IND; 2 Infectious Disease, Jawaharlal Nehru Medical College, Datta Meghe Institute of Higher Education and Research, Wardha, IND

**Keywords:** vaccination programs, multi-drug resistant (mdr), epidemiology, salmonella typhi, antibiotic resistance, enteric fever

## Abstract

Enteric fever, predominantly caused by *Salmonella enterica* serovar Typhi and *Salmonella enterica* serovar Paratyphi, remains a significant global health challenge. This comprehensive review examines the evolving epidemiology and antibiotic resistance associated with enteric fever. We provide an overview of the disease's definition and historical context, highlighting the substantial impact of antibiotic resistance on treatment efficacy. The review details the global burden, incidence trends, and risk factors of enteric fever while elucidating the pathogenesis and clinical manifestations of the disease. A critical analysis of antibiotic resistance mechanisms reveals the alarming rise of multi-drug resistant (MDR) and extensively drug-resistant (XDR) strains, complicating treatment regimens and underscoring the need for novel therapeutic strategies. Current treatment protocols, the role of empirical therapy, and the rational use of antibiotics are discussed in depth. Additionally, we explore prevention and control strategies, emphasizing the importance of vaccination programs, sanitation improvements, and effective public health interventions. The review concludes with recommendations for future actions, including enhanced surveillance, research and development of new antibiotics, expansion of vaccination efforts, and improved public health infrastructure. The findings highlight the necessity for updated clinical guidelines and sustained global efforts to address the challenges of enteric fever and its evolving antibiotic resistance patterns. Through coordinated action and continued innovation, it is possible to mitigate the impact of this enduring public health threat.

## Introduction and background

Enteric fever, also known as typhoid fever, is a systemic infectious disease primarily caused by *Salmonella enterica* serovar Typhi (*S. *Typhi) and, to a lesser extent, by *Salmonella enterica* serovar Paratyphi (*S. *Paratyphi). It is characterized by fever, abdominal pain, and other systemic symptoms, often leading to significant morbidity and mortality if left untreated [1]. The history of enteric fever dates back centuries, with documented outbreaks and descriptions of the disease across different regions. Notable historical figures such as Florence Nightingale and Mary Mallon ("Typhoid Mary") played roles in understanding the transmission and impact of enteric fever. The development of antibiotics in the 20th century revolutionized its treatment but also led to challenges such as antibiotic resistance [2].

Understanding the epidemiology of enteric fever is crucial for effective disease control and prevention strategies. This includes knowledge of its global burden, distribution patterns, incidence trends, and risk factors. Additionally, antibiotic resistance poses a significant threat to the successful treatment of enteric fever, necessitating continuous surveillance and research efforts to combat this issue [3]. This comprehensive review aims to synthesize current knowledge on the evolving epidemiology of enteric fever and the dynamics of antibiotic resistance associated with its causative agents. By examining historical perspectives, recent trends, mechanisms of resistance, treatment options, and prevention strategies, this review seeks to provide insights into the challenges and opportunities in managing enteric fever in the contemporary context.

## Review

Methodology

The methodology employed for selecting articles for this comprehensive review followed a systematic approach to ensure the inclusion of high-quality studies and publications pertinent to the understanding of enteric fever epidemiology and antibiotic resistance. Initially, a thorough search was conducted across electronic databases such as PubMed/MEDLINE, Scopus, Web of Science, and relevant public health repositories, utilizing keywords and Medical Subject Headings (MeSH) terms related to enteric fever, *S. *Typhi, *S. *Paratyphi, epidemiology, antibiotic resistance, treatment, prevention, and control. Articles were included based on their relevance to the review's scope, encompassing studies focusing on the epidemiology of enteric fever, antibiotic resistance patterns, treatment options, prevention strategies, and related public health interventions. Exclusion criteria were applied to filter out articles not written in English, duplicates, conference abstracts without full-text availability, and studies lacking relevance to the specific themes of interest. Titles and abstracts were initially screened, followed by a full-text review to determine final inclusion. Data extracted from selected articles, including study design, population characteristics, key findings, and conclusions, were synthesized thematically to provide a comprehensive overview of the current state of knowledge. By adhering to this rigorous methodology, this review aims to offer a robust synthesis of existing evidence, facilitating a nuanced understanding of the dynamic interplay between enteric fever, antibiotic resistance, and public health interventions.

Epidemiology of enteric fever

Global Burden

According to the findings of the Global Burden of Diseases, Injuries, and Risk Factors Study (GBD) 2017, typhoid and paratyphoid fevers accounted for a substantial burden globally in 2017, amounting to 9.8 million (5.6-15.8) disability-adjusted life-years (DALYs). This marked a notable decrease of 43.0% (35.5-50.6) from 17.2 million (9.9-27.8) DALYs reported in 1990 [4]. The study further projected a decrease in the number of cases, estimating 14.3 million (95% uncertainty interval (UI) 12.5-16.3) cases of typhoid and paratyphoid fevers in 2017, reflecting a decline of 44.6% (42.2-47.0) from 25.9 million (22.0-29.9) cases in 1990 [1]. Age-standardized incidence rates also exhibited a significant decline, plummeting by 54.9% (53.4-56.5) from 439.2 (376.7-507.7) per 100,000 person-years in 1990 to 197.8 (172.0-226.2) per 100,000 person-years in 2017 [1]. Notably, *S. *Typhi was identified as the predominant causative agent, responsible for 76.3% (71.8-80.5) of enteric fever cases in 2017 [4]. The global case fatality rate was estimated at 0.95% (0.54-1.53) in 2017, with higher fatality rates observed among children, older adults, and individuals residing in lower-income countries [4]. This translated to an estimated 135.9 thousand (76.9-218.9) deaths attributed to typhoid and paratyphoid fever globally in 2017, reflecting a decline of 41.0% (33.6-48.3) from 230.5 thousand (131.2-372.6) deaths reported in 1990 [4]. A separate study estimated that 2000 typhoid fever resulted in 21,650,974 illnesses and 216,510 deaths [5]. Geographically, regions with a high incidence (>100/100,000 cases/year) were predominantly located in south-central and southeast Asia. In comparison, areas with medium incidence (10-100/100,000 cases/year) encompassed the remainder of Asia, Africa, Latin America, the Caribbean, and Oceania (excluding Australia and New Zealand). Conversely, Europe, North America, and other developed regions exhibited low incidence rates (<10/100,000 cases/year) [5]. In 2019, modeling data from the Global Burden of Disease study projected an estimated 9.2 million (95% CI = 5.9-14.1) cases of typhoid fever and 110,000 (95% CI = 53,000-191,000) associated deaths worldwide [6]. The highest estimated incidence in 2019 was observed in the WHO Southeast Asian (306 cases per 100,000 persons), Eastern Mediterranean (187), and African (111) regions [6].

Distribution of Enteric Fever

The distribution of enteric fever, caused by *S. *Typhi and Paratyphi A, exhibits considerable variation worldwide, with certain regions bearing a substantial disease burden. Throughout Asia, incidence rates of typhoid fever have been reported at levels ranging from 24 to 493 cases per 100,000 population in countries such as Vietnam, China, Indonesia, Pakistan, and India [7]. The disease maintains an endemic presence across Africa, Asia, Central and South America, the Middle East, and Eastern and Southern Europe [7]. In the United States, cases of enteric fever are primarily linked to travel to southern Asia, where rates of antibiotic resistance are notably high. Notably, between 2008 and 2012, there was a discernible increase in the proportion of paratyphoid A cases, rising from 16% to 22%, with a significant portion of these cases attributed to travel to southern Asia. Furthermore, resistance to nalidixic acid increased during this period, with nearly all nalidixic acid-resistant isolates demonstrating resistance or reduced susceptibility to ciprofloxacin [8]. Various factors, including travel patterns, profiles of antimicrobial resistance, and socioeconomic conditions, influence typhoid fever's geographic distribution. Understanding these distribution patterns is imperative for implementing effective surveillance, prevention, and control measures to combat this infectious disease.

Risk Factors for Enteric Fever

**Figure 1:**
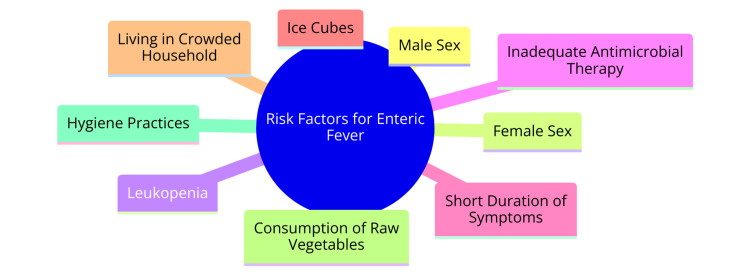
Risk factors for enteric fever Image Credit: Khadija Hamdulay

Several factors have been identified as independent risk factors for enteric perforation in patients with typhoid fever. First, male sex has been consistently associated with a higher risk of perforation, as demonstrated in both univariate and multivariate analyses [9]. Additionally, leukopenia, characterized by a low white blood cell count, has emerged as another significant risk factor for enteric perforation in typhoid fever patients, substantiated by findings from both univariate and multivariate analyses [9]. Inadequate antimicrobial therapy administered before admission has also been linked to an increased risk of perforation in typhoid fever cases, emphasizing the critical role of timely and appropriate treatment [9]. Moreover, a short duration of symptoms has been identified as a risk factor for enteric perforation, underscoring the importance of early recognition and intervention in mitigating complications [9]. Furthermore, specific risk factors have been highlighted in the context of typhoid and paratyphoid fever. Ice cubes have been independently associated with an elevated risk of typhoid fever compared to fever controls, suggesting a potential transmission route through contaminated ice [10]. Conversely, the female sex has been identified as an independent risk factor for typhoid fever in certain studies, particularly when compared with fever controls [10]. Living in crowded households has been associated with an increased risk of typhoid fever in specific regions, emphasizing the role of environmental factors in disease transmission dynamics [10]. Additionally, consuming raw vegetables contaminated with sewage has been independently linked to a higher risk of typhoid fever in specific geographical contexts, highlighting the significance of food safety practices [11]. Finally, poor handwashing hygiene and sharing food from the same plate have been identified as risk factors for the intrahousehold spread of typhoid fever in specific cultural settings, underscoring the importance of hygiene practices in preventing disease transmission [10]. Risk factors for enteric fever are shown in Figure 1.

Pathogenesis of enteric fever

*Causative Agents (*S.* Typhi and *S. *Paratyphi)*

The pathogens responsible for typhoid fever and paratyphoid fever are *S. *Typhi and *S. *Paratyphi, respectively [11]. These bacteria, specifically *S. enterica* serotypes Typhi and Paratyphi A, are the culprits behind these severe systemic infections, each characterized by unique clinical manifestations and transmission methods [11]. Despite causing clinically similar syndromes, *S. *Typhi and *S. *Paratyphi are genetically and phenotypically distinct, exhibiting varying propensities for developing resistance to antimicrobial agents [12]. Both bacteria are primarily transmitted through the fecal-oral route, often via contaminated food or water sources, and are prevalent in regions marked by inadequate sanitation and hygiene standards [12].

Transmission Routes

Typhoid fever, caused by *S. enterica* serovars Typhi and Paratyphi, primarily spreads through the fecal-oral route, typically by consuming contaminated food or water [13-16]. Humans are the sole carriers of these serotypes associated with typhoid fever, with no involvement of animals in the transmission cycle [14]. The bacteria are excreted in the feces of infected individuals and can contaminate water sources or food, facilitating the transmission of the disease upon ingestion by others. This transmission mode underscores the crucial role of sanitation and access to clean water in preventing the spread of typhoid fever [13-16]. In regions with inadequate sanitation and limited access to clean water, such as resource-limited settings, the burden of typhoid fever is notably high due to the heightened risk of fecal-oral transmission from contaminated environments. Outbreaks are more prevalent in these areas and are often linked to various factors, including contaminated food and water sources, street vendors, poor hygiene practices, and insufficient access to proper sanitation facilities [14,15]. Understanding the transmission pathways of typhoid fever is paramount for implementing effective preventive measures, such as enhancing public water and sewage management, promoting hygiene practices, and devising vaccination strategies to mitigate the disease burden in endemic regions [14,15].

Clinical Manifestations

Typhoid fever caused by *S.* Typhi and Paratyphi infections presents with a classic syndrome that is clinically indistinguishable. Symptoms typically manifest 7-14 days following exposure to the bacteria, characterized by a gradual increase in fever. Gastrointestinal symptoms such as abdominal pain, tenderness, and constipation are commonly observed, alongside additional manifestations including a dry cough, headache, delirium, and malaise. Notably, the appearance of Rose Spots, splenomegaly, and relative bradycardia during the second week of illness are characteristic. Severe complications such as bowel perforation, peritonitis, and intestinal hemorrhage may arise in the third week, leading to a typhoid state marked by apathy, confusion, and potentially psychosis [17]. Moreover, typhoid fever can exhibit atypical manifestations, including severe headaches resembling meningitis, acute lobar pneumonia, arthralgias, urinary symptoms, severe jaundice, or neurologic manifestations like delirium, parkinsonian symptoms, or Guillain-Barré syndrome. In certain regions like India and Africa, patients may primarily present with neurologic symptoms [18]. The clinical course of untreated typhoid fever may vary depending on geographic region, racial factors, and the infecting bacterial strain. While the classic stepladder fever pattern, once prevalent, now occurs in a minority of cases, young children, individuals with AIDS, and a significant proportion of immunocompetent adults may experience diarrhea instead of constipation [18]. Additionally, typhoid fever can lead to various complications, such as anemia, gastrointestinal bleeding, bone marrow hypoplasia, encephalopathy, disseminated intravascular coagulation, and shock. Although the mortality rate significantly decreases with treatment, some patients may become asymptomatic carriers after recovery, continuing to shed the bacteria [19].

Complications

Gastrointestinal bleeding, a complication of enteric fever, may arise from ulcers in various gastrointestinal tract sections, including the terminal ileum, ileocecal valve, ascending colon, and transverse colon [20,21]. Symptoms indicative of gastrointestinal bleeding encompass fatigue, breathlessness, pallor, irregular heartbeat, vomiting of blood, and the passage of dark or tar-like stool [18]. Intestinal perforation represents a critical complication wherein a breach in the intestinal wall leads to the leakage of contents into the abdominal cavity, resulting in severe abdominal pain, vomiting, and potentially sepsis [20,21]. This condition necessitates urgent medical attention, often requiring prompt administration of antibiotics and surgical intervention to repair the perforation [18]. Furthermore, Salmonella infection can induce cholestatic hepatitis, characterized by jaundice, hepatomegaly, and abnormalities in liver function tests [19,20]. Other potential complications encompass myocarditis, inflammation of the heart muscle, as well as encephalopathy, which manifests as confusion, delirium, and other neuropsychiatric symptoms [19,20]. Additionally, pneumonia may develop as a secondary complication of enteric fever, further complicating the clinical course [19,20]. Acute kidney injury represents another potential complication, with the dysfunction of renal function being observed in some cases [19]. Chronic carriage of the bacteria occurs in a small percentage of patients, ranging from 2-5%, wherein the bacteria persist in the gallbladder or other sites, leading to asymptomatic carriage and continued shedding [19]. Complications are more likely in patients not promptly treated with appropriate antibiotics [19]. Intestinal perforation, in particular, poses a severe threat, with mortality rates reaching up to 20% in certain studies [19,20]. Hence, timely diagnosis, antibiotic treatment, and the management of complications are imperative to mitigate morbidity and mortality associated with enteric fever.

Antibiotic resistance in enteric fever

Mechanisms of Antibiotic Resistance

Antibiotic resistance mechanisms employed by bacteria encompass various strategies to neutralize the effects of antimicrobial agents. First, bacteria can undertake inactivation processes wherein they physically dismantle antibiotics before the drugs can exert their therapeutic effects, thereby rendering the antibiotics ineffective [22]. Another mechanism involves altering the binding sites targeted by antibiotics, preventing the drugs from binding and effectively exerting their intended actions [23]. Additionally, some bacteria can evade the activity of antibiotics by altering metabolic pathways, enabling them to synthesize essential compounds in alternative ways, thereby circumventing the effects of the antibiotic [24]. Moreover, bacteria can employ mechanisms to diminish the accumulation of antibiotics within their cells, thereby reducing the intracellular levels of the drugs. These mechanisms may involve reducing membrane permeability or deploying efflux pumps that actively pump the antibiotics out of the bacterial cell, thereby compromising the efficacy of the antibiotic [25]. These adaptive mechanisms underscore bacteria's resilience in developing antibiotic resistance, presenting significant challenges in effectively treating infections. Understanding these resistance mechanisms is imperative for devising strategies to counter antibiotic resistance and enhance the efficacy of antimicrobial treatments [25].

Historical Trends in Antibiotic Susceptibility

One study analyzed the antibiotic susceptibility patterns of methicillin-resistant *Staphylococcus aureus* (MRSA) isolates across several hospitals in Riyadh, Saudi Arabia, from 1994 to 2002 [1]. The findings of the study indicated that quinupristin/dalfopristin and linezolid exhibited the highest efficacy against MRSA; however, resistance to these antibiotics had already emerged during the study period, underscoring the necessity of restricting their use to preserve their effectiveness [26]. Another review delved into the origins and evolution of antibiotic resistance, highlighting the emergence of resistant bacterial strains in tandem with the discovery and clinical implementation of antibiotics [27]. Notably, shortly after the introduction of streptomycin in 1944 for tuberculosis treatment, resistant strains of Mycobacterium tuberculosis were observed during patient treatment [27]. Additionally, a study addressing antimicrobial resistance among anaerobic bacteria observed a global increase in resistance rates, varying across geographic regions and often between species [28]. The review stressed the critical role of susceptibility testing in guiding appropriate antibiotic therapy in light of escalating resistance levels [28]. Meanwhile, a study conducted in a newly constructed cancer hospital in India aimed to assess microbial and antibiotic resistance patterns in clinical samples [29]. The study revealed a notably high percentage of resistance among organisms to beta-lactam antibiotics and combination beta-lactam/beta-lactamase inhibitors, alongside a prevalent occurrence of extended-spectrum beta-lactamase (ESBL) production among Gram-negative bacteria [29].

Current Status of Antibiotic Resistance

Antibiotic resistance presents a pressing global health challenge, with bacteria resistant to antibiotics posing a significant threat to public health worldwide. The misuse and overuse of antibiotics have spurred the rapid emergence of resistant strains, undermining the effectiveness of these crucial medications [30]. Annually, antibiotic-resistant bacteria contribute to over 1.27 million deaths, a figure projected to escalate substantially without urgent intervention [31]. This alarming reality has ushered in what is termed a "post-antibiotic era," where existing antibiotics increasingly fail to combat bacterial infections due to the development of resistance [30]. This crisis is not merely a future concern but an immediate reality, carrying profound implications for healthcare systems and populations globally [30]. The rise of multi-drug resistant (MDR) and extensively drug-resistant (XDR) bacteria, notably gram-negative pathogens, has further compounded treatment challenges reminiscent of a bygone pre-antibiotic era [30]. Mitigating the antibiotic resistance crisis demands concerted efforts, including developing novel antimicrobial therapies, repurposing existing drugs, and leveraging mathematical prediction models to bolster treatment strategies and confront the scourge of antimicrobial resistance [30]. Coordinated actions, revitalized research endeavors, and effective management protocols are imperative to mitigate the adverse impact of antibiotic resistance on public health and healthcare systems [30].

Factors Contributing to Antibiotic Resistance

The factors contributing to antibiotic resistance are complex and encompass various elements, including the overuse and misuse of antibiotics, incorrect diagnoses, the absence of rapid infection tests, and insufficient adherence to recommended behaviors such as completing antibiotic courses [32]. Genetic factors also significantly influence antibiotic resistance, with intrinsic and acquired bacterial genes playing pivotal roles. Mechanisms such as β-lactamases, carbapenemases, and extended-spectrum β-lactamases (ESBLs), as well as resistance genes targeting diverse antibiotic classes like glycopeptides, macrolides, and fluoroquinolones, are significant contributors [33,34]. Furthermore, the extensive use of antibiotics in human and animal health, including their utilization for animal growth promotion, has been a primary driver of antimicrobial resistance. This practice has facilitated the acquisition of resistance determinants by bacteria across various environments and has fostered the dissemination of antibiotic-resistant bacteria and genes [34,35]. The misuse of antibiotics, both in human and animal contexts, emerges as a significant determinant of antimicrobial resistance, with factors such as suboptimal dosing, limited access to diagnostics, and environmental contamination further exacerbating resistance development [32]. Comprehending these multifaceted factors is paramount to addressing the global challenge of antibiotic resistance. It underscores the importance of promoting responsible antibiotic usage, implementing practical diagnostic tools, and enhancing public awareness to combat the emergence and dissemination of resistant bacteria effectively.

Treatment of enteric fever

Empirical Therapy

Empirical therapy for enteric fever, encompassing both typhoid and paratyphoid fever, entails the administration of antibiotics based on the patient's clinical presentation and the likelihood of the infection being caused by either *S. *Typhi or* S.* Paratyphi [36]. The selection of empirical therapy is guided by the severity of the illness and the presence of any associated complications. In cases of severe illness or hospitalized patients with complications, ceftriaxone is recommended as the first-line treatment, with cefotaxime or aztreonam serving as alternative options [36]. For outpatient management, cefixime is the preferred first-line choice, with azithromycin considered a second-line option, particularly in scenarios with a documented penicillin allergy [36]. The use of steroids is reserved for severe cases of enteric fever, mainly when patients exhibit signs of shock, coma, or altered sensorium [36]. Dexamethasone may be administered at a dosage of 3 mg/kg followed by 1 mg/kg every six hours for two days. However, prolonged steroid use can elevate the risk of relapse and induce adverse effects; therefore, their utilization should be judicious [36]. It is imperative to emphasize that antimicrobial sensitivity results must guide the treatment of enteric fever, as these are instrumental in determining the most appropriate antibiotic therapy [36]. The emergence of antimicrobial resistance, notably toward fluoroquinolones, has significantly complicated the management of enteric fever. In cases of resistance, alternative antibiotics such as azithromycin and carbapenems may be necessary to achieve therapeutic efficacy [37,38]. This underscores the importance of ongoing surveillance of antimicrobial resistance patterns and the judicious use of antibiotics to mitigate the escalation of resistance and optimize patient outcomes.

Antibiotic Options

The treatment landscape for enteric fever, encompassing both typhoid and paratyphoid fever, has undergone significant evolution due to the emergence of multidrug resistance to first-line agents like amoxicillin/ampicillin, cotrimoxazole and chloramphenicol [39]. The subsequent reliance on fluoroquinolones was challenged by the rise of intermediate and total fluoroquinolone resistance, particularly prominent in South Asia since the late 1990s [39]. Consequently, extended-spectrum cephalosporins such as ceftriaxone (administered intramuscularly or intravenously) and cefixime (given orally), along with azithromycin (an oral macrolide), have emerged as preferred treatment options for fluoroquinolone-resistant isolates [38,39]. Ceftriaxone is noted for its effectiveness in treating enteric fever with minimal adverse effects, and it stands alongside azithromycin, fluoroquinolones, and chloramphenicol in its efficacy against the disease [39]. Azithromycin and gatifloxacin are comparably effective as oral agents, while ofloxacin has become ineffective even at higher doses due to prevailing resistance [38]. Ceftriaxone is recommended as a primary treatment for enteric fever, particularly in instances of antibiotic resistance [38,39]. Alongside antibiotic therapy, supportive measures such as fluid intake to prevent dehydration stemming from fever and diarrhea are essential, with surgical intervention occasionally warranted in severe cases to rectify intestinal damage [36]. Completing the entire antibiotic course as prescribed by a healthcare provider is crucial to mitigating the risk of antibiotic resistance [40]. Preventive strategies, including vaccination before traveling to high-risk areas for typhoid fever and adherence to good hygiene practices, are pivotal in disease management [40]. Dietary adjustments, such as consuming easily digestible foods like cooked vegetables, refined grains, and low-fat or fat-free milk, are recommended to alleviate symptoms while avoiding spicy foods and those high in fiber and fat [40]. These multifaceted approaches are integral in the comprehensive management of enteric fever, ensuring effective treatment outcomes and prevention of disease transmission.

Rational Use of Antibiotics

The rational use of antibiotics is a cornerstone in healthcare, striving to achieve optimal treatment outcomes while mitigating toxicity and curbing the development of antimicrobial resistance. Fundamental principles contributing to this endeavor include avoiding unnecessary antibiotic prescriptions, ensuring accurate dosing, adhering to appropriate treatment durations, and maintaining suitable dosage intervals [41,42]. Antibiotics should be administered solely for documented infections, and it is imperative to complete the entire prescribed course of treatment to forestall the emergence of resistance [42]. Globally, efforts to promote rational antibiotic use have gained traction, with initiatives like the European Awareness Day for Rational Use of Antibiotics, observed on November 18, aimed at raising awareness among the public and healthcare practitioners to curtail antimicrobial overuse through informed prescribing practices [42]. The research underscores the ramifications of irrational antibiotic use, including the escalation of antimicrobial resistance, treatment failures, and heightened mortality rates, particularly evident in critical care settings [41]. Studies conducted at institutions like the Kenyatta National Hospital reveal prevalent instances of inappropriate antibiotic selection and incorrect treatment durations, underscoring the imperative for enhanced adherence to antibiotic prescribing guidelines to bolster patient outcomes and reduce mortality rates [41]. Given antimicrobial resistance's profound health and economic implications, fostering rational antibiotic utilization is paramount in global health agendas. Strategies encompassing education initiatives to enhance understanding and attitudes toward antibiotic usage and policy interventions aimed at regulating antibiotic sales and fostering judicious prescribing practices are pivotal in fostering rational antibiotic use behavior across healthcare settings and within the broader populace [43].

Challenges in Treatment

The treatment landscape for enteric fever, encompassing both typhoid and paratyphoid fever, confronts significant hurdles owing to the emergence of multidrug resistance to first-line agents such as amoxicillin/ampicillin, cotrimoxazole, and chloramphenicol [40,44]. This resistance has propelled the utilization of fluoroquinolones; however, since the late 1990s, intermediate and total fluoroquinolone resistance has surfaced, particularly prevalent in South Asia [40]. Consequently, treatment with extended-spectrum cephalosporins like ceftriaxone (administered intramuscularly or intravenously) and cefixime (taken orally) or azithromycin (an oral macrolide) often emerges as the preferred course of action for fluoroquinolone-resistant isolates [40]. Local resistance patterns and susceptibility testing should guide the selection of antibiotics for enteric fever [16]. In certain regions, combining fluoroquinolone with azithromycin or third-generation cephalosporin with azithromycin has become a recommended approach [16]. However, the escalating resistance to newer antibiotics poses a concerning challenge, complicating the management of enteric fever [16]. Chronic carriers require treatment with four weeks of ciprofloxacin or fluoroquinolones [45]. For patients experiencing relapse, infection by the same strain mandates an extended antibiotic therapy duration [45]. In India, delayed clinic presentations, inadequate diagnostic facilities, and suboptimal test usage impede the effective control of enteric fevers [3]. Pre-exposure to inadequate and unnecessary antibiotic therapy before seeking proper healthcare may compromise the performance of diagnostic tests, thereby influencing subsequent patient management concerning antimicrobial resistance and serious complications [3]. The escalating prevalence of resistance to available antibiotics translates into heightened morbidity, mortality, and treatment costs [3]. The emergence of multidrug resistance in *S. *Typhi has emerged as a prominent concern in India, marked by chloramphenicol resistance outbreaks in 1972, succeeded by amoxicillin, co-trimoxazole, and chloramphenicol resistance by the 1990s [3]. Ciprofloxacin resistance emerged in the late 1990s [3]. Physicians often prescribe azithromycin or cefixime for uncomplicated cases. At the same time, intravenous ceftriaxone therapy is recommended as per the National Treatment Guidelines for Antimicrobial Use in Infectious Diseases issued by the National Centre for Disease Control [3]. Nonetheless, the burgeoning resistance leading to heightened disease severity, morbidity, and mortality underscores the imperative of meticulous monitoring, surveillance, and case reporting to avert the necessity for last-line antimicrobials in therapy [3].

Prevention and Control Strategies

Vaccination programs: Various vaccines are utilized for typhoid fever in areas of prevalence or outbreak. These include the injectable Vi polysaccharide vaccine (ViPS), oral Ty21a vaccine, and injectable typhoid conjugate vaccine (TCV) [46]. Typhoid conjugate vaccines (TCV) have exhibited high efficacy, offering prolonged protection and suitability for young children. They have demonstrated promising outcomes in thwarting typhoid fever, including infections stemming from antibiotic-resistant strains [47]. The World Health Organization (WHO) advocates incorporating typhoid conjugate vaccines into routine immunization programs in endemic countries. Since December 2017, two TCVs have been prequalified by WHO, with numerous nations integrating them into childhood vaccination initiatives [48]. Vaccination programs adopt various strategies based on disease control objectives (preemptive for endemic disease or outbreak prevention and reactive for outbreak management) and vaccine distribution tactics (community-based routine, community-based campaign, and school-based). These approaches have proven acceptable, feasible, and efficacious across diverse contexts, with the most robust evidence of impact observed in endemic areas [46]. Organizations such as the CDC collaborate with global partners and countries to endorse the utilization of typhoid fever vaccines in outbreak situations and national immunization schemes. Their endeavors encompass documenting the typhoid fever burden, facilitating vaccine introduction, assessing optimal vaccination practices, and addressing impediments to vaccination in endemic regions [47]. In the UK, two primary vaccines are available to combat typhoid fever: the Vi vaccine (administered via a single injection) and the Ty21a vaccine (consumed as three capsules taken on alternate days). These vaccines elicit antibody production within the body, averting illness upon exposure to typhoid bacteria. Booster vaccinations are recommended every three years to sustain ongoing protection [18].

Sanitation and hygiene measures: Access to improved sanitation facilities significantly lowers the risk of contracting typhoid fever compared to using unimproved facilities, which do not prevent human contact with excreta. Improved facilities such as flush toilets or pit latrines are associated with reduced odds of typhoid fever transmission [49]. Basic hygiene practices, including handwashing with soap and water, are pivotal in preventing typhoid fever. Having a handwashing facility with soap available at home can decrease the odds of typhoid fever by 40%, highlighting the importance of this simple yet effective measure [50]. Handwashing with soap is recognized as a critical preventive measure against diarrheal diseases, including typhoid [48]. Safe food handling practices are crucial in preventing typhoid transmission through contaminated food. It involves ensuring that food is thoroughly cooked, avoiding raw milk and products derived from it, and meticulously washing fruits and vegetables before consumption [48]. Safe water storage practices are also essential to reduce the risk of contracting typhoid fever from contaminated water sources. Storing drinking water in clean containers and treating water before consumption, either by boiling or using a reliable disinfectant, can help mitigate the risk of typhoid transmission [48]. Proper waste disposal is imperative for preventing the spread of typhoid bacteria through contaminated soil or water sources. Safely disposing of human waste and maintaining a clean environment around the home are fundamental measures in minimizing the transmission of typhoid fever [3]. These practices collectively contribute to effective prevention strategies against typhoid fever, emphasizing the importance of hygiene, sanitation, and safe food and water practices in reducing the burden of this infectious disease.

Surveillance and monitoring*: *Environmental surveillance (ES) is a cost-effective tool for identifying communities with a high burden of typhoid fever. This method employs standardized sampling, validation, and characterization protocols, utilizing quantitative PCR to detect Salmonella genes and markers of human fecal contamination. ES can delineate the prevalence and distribution of *S. *Typhi, even in the absence of clinical cases, furnishing crucial data for decision-making and vaccine introduction [51]. National Surveillance Systems, administered by the Centers for Disease Control and Prevention (CDC) in the United States, oversee surveillance for typhoid fever and paratyphoid fever through various platforms such as the National Notifiable Diseases System, Laboratory-based Enteric Disease Surveillance System, National Antimicrobial Resistance Monitoring System (NARMS), National Outbreak Reporting System, and Foodborne Diseases Active Surveillance Network. These systems gather and consolidate data on laboratory-confirmed cases, antimicrobial resistance, outbreaks, and more, facilitating the determination of incidence and trends and guiding prevention efforts [52].

Global Surveillance Studies, including initiatives like the Surveillance for Enteric Fever in Asia Project (SEAP), Severe Typhoid Fever Surveillance in Africa program (SETA), and Surveillance of Enteric Fever in India (SEFI), have been established to estimate age-specific incidence of enteric fever, assess disease burden, and inform vaccination strategies. These studies seek to furnish data on disease burden across diverse settings, elucidate clinical features, and advise on the optimal use of typhoid conjugate vaccines [52]. The utility of surveillance studies such as ES and serological surveillance lies in their ability to capture both symptomatic and asymptomatic infections, providing a sensitive approach independent of healthcare-seeking behavior. However, challenges persist, including the necessity for continuous surveillance, validation of alternative low-cost methods, and ensuring the sustainability of surveillance programs to monitor changes in disease incidence over time [52].

## Conclusions

In conclusion, this comprehensive review has elucidated the significant challenges and evolving landscape of enteric fever, mainly focusing on its epidemiology and the alarming rise of antibiotic resistance. The findings highlight the global burden of the disease, the complexities introduced by MDR and XDR strains, and the implications these have for clinical practice and public health. Updated clinical guidelines, robust surveillance systems, enhanced vaccination programs, improved public health infrastructure, and increased public awareness are essential in combating this public health threat. Moving forward, sustained investment in research and development, alongside coordinated global efforts, will be crucial in managing and ultimately eradicating enteric fever, ensuring better health outcomes for affected populations worldwide.
